# C. G. Jung’s Concept of “Manic Mood” From 1904: An Early Contribution to the Disorder of the Adult Form of ADHD?

**DOI:** 10.1177/10870547251319077

**Published:** 2025-02-22

**Authors:** Steffen Müller, Maria Strauß, Holger Steinberg

**Affiliations:** 1 Department of Psychiatry and Psychotherapy, University of Leipzig Medical Center, Leipzig, Germany; 2 Research Center for the History of Psychiatry, Department of Psychiatry and Psychotherapy, Medical Faculty of the University of Leipzig, Leipzig, Germany

**Keywords:** adult ADHD, adult, history of psychiatry, Carl Gustav Jung, chronic mania

## Abstract

**Background:**

Adult ADHD has increasingly become a focus in adult psychiatry. Despite well-established diagnostic criteria and specific therapeutic approaches, contemporary discussions often dismiss ADHD as a “fad.” This study examines Carl Gustav Jung’s 1904 concept of “manic mood” and its potential alignment with the modern understanding of ADHD in adults.

**Objective:**

The aim of this paper is to investigate and discuss whether Jung’s concept of “manic mood” can be considered part of the intellectual history of adult ADHD.

**Method:**

Jung’s concept of “manic mood” is analyzed and presented using the literary-historical method of “close reading,” placing the analysis within the context of the early 20th-century discussion of “chronic-manic concepts.” This analysis is compared with the current diagnostic criteria for adult ADHD.

**Results and Conclusion:**

Jung’s concept of “manic mood,” described in 1904, has clear parallels to diagnostic criteria used for adult ADHD. It is conceivable that the patients presented by Jung would be diagnosed with adult ADHD today. Jung’s work fits into the discussion of “chronic-manic concepts” of the early 20th century and thus makes a relevant original contribution to the nosological-diagnostic classification of forms in the spectrum of ADHD-affective disorders-personality disorders. The parallel between Jung’s disease construct of “manic mood” and the current view of ADHD supports our hypothesis that the adult form of ADHD represents a consistent disease phenomenon and should therefore not be degraded as a “fad.”

## Introduction

ADHD in adulthood has become increasingly relevant for adult psychiatry since the 1980s, with a current estimated prevalence of 2.5% to 6.2% in the general population (Fayyad et al., 2017). Once thought to be a disorder that occurs exclusively in childhood, research now indicates that up to 60% of children with ADHD continue to experience symptoms into adulthood (Fayyad et al., 2007). Recent decades have seen an increase in diagnosis rates, which likely reflects improvements in recognition and diagnostic practices driven by the expansion of specialized ADHD outpatient clinics and a growing understanding of the disorder within general psychiatric care (Xu et al., 2018). Despite this progress, many clinicians outside of specialized settings remain hesitant to treat adults with ADHD, often dismissing it as a “trend” or “fad” (Bisset et al., 2022).

While adult ADHD was first listed in the Diagnostic and Statistical Manual of Mental Disorders (DSM)—III in 1980 under the diagnostic group of “Attention Deficit Disorder Residual Type” (ADD-RT), it seems relevant to examine to what extent descriptions of ADHD-specific symptoms can be identified in psychiatric publications published before then. In contrast to the historical analysis of the childhood form of ADHD, the psychiatric-historical investigation of the existence or non-existence of the adult form of this disorder still appears incomplete.

In his overview of the historical development of ADHD, Conner (2000) outlines the key milestones in the history of ADHD, focusing on pediatric ADHD. In other descriptions of the history of adult ADHD (Barkley, 2015; Barkley & Peters, 2012), the German physician Melchior Adam Weikard (1742–1803) is acknowledged as the first person to describe the disorder. In 1775, Weikard reported on characteristic symptoms of ADHD in his patients in an anonymously published paper (Weikard, 1775). More recently, attention has been drawn to the early medical description of an attention disorder by the Dutch physician Cornelius Kloekhof (1715–1788) in 1753, which currently represents what is perhaps the earliest mention of ADHD-like symptoms (Van Kernebeek & Crunelle, 2024). The Scotsman Alexander Crichton (1763–1856), who published his own observations on the symptoms of motor restlessness, behavioral abnormalities, and concentration disorders in 1798, is also mentioned. The London pediatrician George Still (1868–1941), who made the disorder accessible to a wider medical audience for the first time in 1902, is considered groundbreaking for the understanding and conceptualization of ADHD in children (Still, 1902).

After these early empirical descriptions of ADHD-typical symptoms, ADHD in adults increasingly became the focus of neuropsychiatric research from the 1960s onwards (Lange et al., 2010). The lack of a uniform conceptualization over many years has resulted in debates regarding the understanding and acceptance of this disorder that continue to this day. Although ADHD-specific symptoms have arguably been described in medical literature for more than 250 years, the operationalization of the disorder in internationally used classification systems DSM and ICD did not take place until much later. This suggests that those affected were previously categorized under other diagnostic entities.

Recent studies have demonstrated significant parallels between the nosological construct of “chronic mania,” which was actively discussed by prominent neurologists in the German-speaking world in the early 20th century, and the contemporary concept of ADHD (Müller et al., 2024; Steinberg & Strauß, 2020, 2022). At the core of this debate was the question of the existence or non-existence of the disorder known as “chronic mania.” There was no general consensus on this issue. Some of the authors involved in the discussion vehemently doubted the very existence of chronic mania as a distinct disorder, while others speculated that it represented an independent disease entity. Among the former representatives of the psychiatric community who dealt with the topic were Emil Kraepelin (1856–1926), Carl Wernicke (1848–1905), Eugen Bleuler (1857–1939), and Gustav Specht (1860–1940; Table 1). They described “chronic-manic concepts” through case discussions about adult patients, many of whom would probably be diagnosed with ADHD today, characterizing it as a disorder that existed along a continuum between healthy and ill with symptoms persisting for years or even a lifetime. In the context of daily demands from school, work, and social life, it appeared that individuals suffering from “chronic mania”—much like those diagnosed with ADHD today—struggled with their symptoms and experienced significant distress as a result. Some of the authors at the time classified this disorder within the broader field of affective disorders or “manic-depressive insanity,” which was defined more broadly than it is today. Others, however, viewed it as a variation of psychopathy. Hereditary factors were considered particularly significant in its etiology. Psychiatrists at that time already had difficulties classifying this disorder into existing nosological schemes, as shown by Müller et al. (2024). However, their models show many similarities to the diagnostic criteria commonly used today for adult ADHD.

**Table 1.** Overview of the Main Protagonists in the Debate Surrounding “Chronic Mania” in the German-speaking World in the Early 20th Century (Müller et al., 2024).

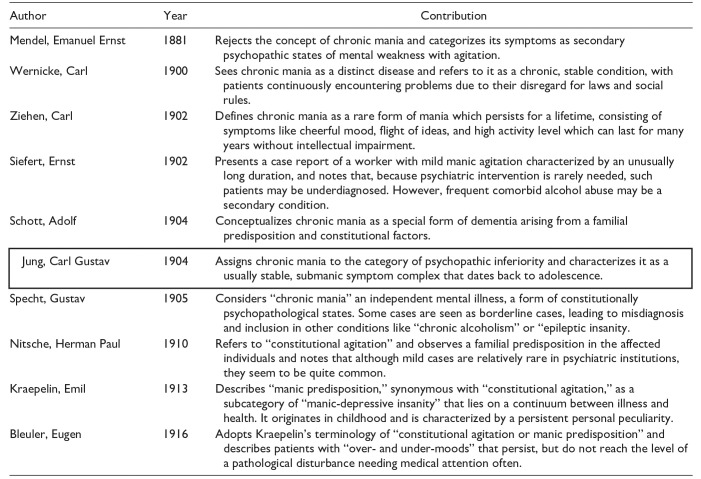

*Note*. The table provides a summary of the respective concepts of “chronic mania,” along with the contributions of the respective authors, and is based on Müller et al. (2024).

While the concept of “chronic mania” has remained under-researched and has faded into obscurity over time, comparative studies, have identified additional parallels with other psychiatric disorders (Gambogi et al., 2016).

*Carl Gustav Jung* (1875–1961), at the time a 29-year-old Zurich neurologist, later a disciple of Freud and founder of analytical psychology, also took part in the discussion about “chronic mania” with great enthusiasm (Jung, 1904). His innovative contribution to this discourse has not been extensively discussed to date (Müller et al., 2024). Assuming that the adult form of ADHD, as described in the psychiatric literature, is not a “fad diagnosis,” but a consistent concept, it seems particularly relevant to meticulously pursue possible historical evidence and contextualize these discussions, both longitudinally and cross-sectionally.

## Method

The early contribution of C. G. Jung (1904) was analyzed using the literary and historical method of “close reading,” while contextualizing the analysis as part of the discussion of “chronic-manic concepts” in the early 20th century.

The 24-page article “About manic mood” (Original: Über manische Verstimmungen) was published in 1904 in the “Allgemeine Zeitschrift für Psychiatrie und psychisch-gerichtliche Medizin,” the most widely circulated German-language psychiatric journal at the time. The contents of this work were analyzed and contrasted with currently valid diagnostic manuals for adult ADHD. As part of the investigation and discussion, it was determined to what extent the concept of “manic mood” can be interpreted as part of the history of adult ADHD and whether it can be used to fill a gap in knowledge about the conceptual development of adult ADHD. A preliminary cursory review of C. G. Jung’s complete works revealed no other writings that address the topic of “manic mood” with comparable depth. Therefore, we contextualized the work within Jung’s complete publication record to introduce his thinking and working world, enable a comprehensive historical contextualization, and appropriately acknowledge Jung’s contributions.

## Contextualization

Carl Gustav Jung is considered one of the most influential psychiatrists and psychologists of the 20th century. The founder of analytical psychology, at times *regarded* as the *crown prince of* Sigmund *Freud, Jung is known worldwide for his theories of archetypes, the collective unconscious, synchronicity, and individuation.* In addition to his pioneering work in the field of depth psychology, Freud’s congenial partner was already well-known during his lifetime due to his professional and private relationships with his patients Toni Wolff (1888–1953) and the Russian psychoanalyst Sabina Spielrein (1885–1942). Furthermore, his correspondence with Hermann Hesse, who later won the Nobel Prize for Literature, still reveals the image of a multi-layered character who has already been examined in detail in numerous works and is still of great interest today (Bair, 2003; Hannah, 1997; Jung & Jaffé, 1984; McLynn, 1997).

Jung laid the foundations of his intellectual legacy in 1900 when he joined the Burghölzli, the university psychiatric hospital in Zurich (Switzerland), as a young assistant under the direction of Eugen Bleuler (1857–1939). Jung was well aware of the discussion of “chronic-manic concepts” at the time. In later comments on this early period of psychiatry at Burghölzli, Jung was perplexed by what he saw as the inadequate role of psychology in the diagnosis and treatment of psychiatric illnesses.

He writes: *“Psychiatric doctrine was designed to abstract from the personality of the sick, so to speak, and to rather make do with diagnoses, descriptions of symptoms and statistics. From the so-called clinical point of view, which prevailed at the time, doctors were not concerned with the mentally ill person as a human being, as an individual, but treated patient no. X. as the host of a long list of diagnoses and symptoms. They <<labeled>> him, stamped him with a diagnosis, and that was the end of the case for the most part. The psychology of the mentally ill person played no role at all”* (Jung & Jaffé, 1984, p. 121).

Jung received his doctorate in 1902 and, in the years that followed, engaged extensively with the works of Sigmund Freud, which had a lasting influence on his career. His first personal correspondence with Freud took place in 1907.

The work of Jung which is this essay’s focus falls into the period of early psychiatric discovery which was still largely uninfluenced by his later exchanges with Freud. Jung was well informed about the “psychiatric mentality” of the early 20th century through his study of the scientific literature of his time and his work under Eugen Bleuler. It is therefore not surprising that the young researcher took part in the discussion of the concepts of “chronic mania” at the time.

## Results

Jung’s essay begins with a declaration of his intention to publish some cases of illness whose peculiarity consisted of “a chronic submanic behavior” and which he now wanted to present under the name of “manic mood” (Jung, 1904, p. 15). He refers to newly published cases which, in his opinion, “still belong entirely to the area of psychopathic inferiority” and are characterized by a “sanguine temperament” (Jung, 1904, p. 15). In this context, he mentions, among others, the psychiatrist Ernst Siefert (1874–1940) from Halle (Germany), who described a chronic manic state that could be traced back to adolescence for the first time (Jung, 1904, p. 15; Siefert, 1902). According to Jung, earlier authors had only hinted at similar cases, but these were insufficiently documented and too broadly defined. The situation was different with the Wroclaw professor Carl Wernicke (1838–1905), who was convinced that acute mania could never turn into a chronic condition. He therefore indirectly described “chronic mania” as an independent entity, even if he could not make precise statements about its origin. Jung then quotes verbatim from Wernicke’s “Grundriss der Psychiatrie”: chronic mania has “all the essential characteristics of acute mania, just modified in such a way as the conditions of a chronic, yet stable state entail. The flight of ideas therefore remains within moderate limits and is still under the influence of a certain level-headedness and self-control. Accordingly, the cheerful mood is not very pronounced, but does occasionally break through.” Accordingly who suffered from this condition would continuously get into trouble by disregarding laws and social rules (Jung, 1904, p. 16; Wernicke, 1900, pp. 369–370).

While agreeing with Wernicke’s description in principle, Jung expresses reservations toward adopting it entirely, as the aforementioned construct is still “too broad” for him. After all, a large number of “unstable people in Magnan’s sense, many troublemakers and morally imbeciles (morally insane)” could be subsumed under this condition (Jung, 1904, p. 17). In order to make the diagnosis of a “chronic submanic state” (Jung, 1904, p. 17) Jung argues that the cardinal symptoms of mania are required, since the sole presence of an occasionally “cheerful mood, overestimation of one’s own abilities, mental productivity, [and] collisions with the law” (Jung, 1904, p. 17) would not suffice. In order to arrive at an accurate diagnosis, “above all the cardinal symptoms of mania are required: emotional instability with predominantly cheerful moods, flight of ideas, distractibility, over-busyness (or urge to move) and, depending on the main symptoms: overestimation of self, ideas of grandeur, alcoholism and other moral defects” (Jung, 1904, p. 17).

Jung states that he considers the term “chronic mania,” as defined by Wernicke and Siefert, to be “too strong,” as in his opinion it is not an independent mania, but rather a submanic state. He personally preferred the term “constitutional manic mood” (Jung, 1904, p. 18) to describe not a “real mania,” but rather a “submanic state,” often mixed with other “psychopathic traits” (Jung, 1904, p. 18). This was ultimately also a reason for “drawing the boundaries of [this] clinical picture as narrowly as possible and always demanding the existence of the core symptoms of mania” (Jung, 1904, p. 18). In the following, Jung attempts to illustrate his concept of “manic mood” using four different case histories. Two cases are mentioned here as examples.

### Case 1

In his first case report, Jung describes a 29-year-old merchant who had become conspicuous due to a “mild form of manic mood” and was suffering from “simple psychopathic instability” (Jung, 1904, p. 18). Although he had been a bright and intelligent child, he had already presented himself as “absent-minded and inattentive” at school, according to his own account, and had also always engaged in “allotria.” Later, he was “lazy and superficial,” showed no perseverance at work, but was capable of “great achievements” as long as he made an effort. During his education, he had allowed himself to be “distracted from work by all sorts of amusements” and had repeatedly turned to alcohol (Jung, 1904, p. 18). In addition, the patient was noted for frequent “moodiness” and a certain impulsiveness. He was often restless, disregarded the authority of his superiors and led a “licentious lifestyle with excesses in every respect” (Jung, 1904, p. 19). In addition, the patient had repeatedly attracted the attention of those around him with his mood swings:

“During these [mood swings] he was disgruntled for no reason, irritable at times, at times depressed, had gloomy thoughts, pessimistic views of the world, or was so irritable that when his mother or sister asked him something, he had to stop himself from hitting the table ‘with both fists’. He could not concentrate on any work at all, being constantly tormented by ‘a terrible inner restlessness’’, a ‘restless eternal urge to get away’, to change his situation, prevented him from doing anything worthwhile” (Jung, 1904, p. 19).

After the patient was admitted to Burghölzli on July 22, 1901, Jung described the patient as particularly “lively” and “talkative.” He was conspicuous for his “blasé judgment,” which was “never thorough.” The patient continued to “getting carried away” and stood out because he started a large number of things but never finished them (Jung, 1904, p. 20).

*He purchased over 100 books within a short period of time, not even reading half of them. His room was filled with newspapers, funny papers, picture postcards and photographs. He took drawing lessons and showed off his artistic talent. After 3 or 4 lessons he gave up drawing, and the same went for riding lessons (Jung, 1904, p. 20).*

In his differential diagnostic considerations, Jung noted that, despite the patient’s frequent excessive drinking, “ alcoholism as such . . . was certainly not present, since the mental abnormality had persisted unchanged, even during the period of abstinence” (Jung, 1904, p. 21). For him, a distinction had to be made between “psychopathic instability” and the “manic mood” mentioned in the case. Jung considered the latter to be more relevant for the diagnosis due to the “minor flight of ideas,” the “predominantly cheerful and completely inadequate mood,” and the “busyness without consistency and perseverance” (Jung, 1904, p. 21).

### Case 3

In another case study, Jung presents the case of the nurse Miss C., who was particularly known for her “social instability” (Jung, 1904, p. 25). Jung was impressed by the “extraordinary inconstancy and restlessness of the patient,” who had “changed her position 32 times in eleven years” (Jung, 1904, p. 28). According to his descriptions, this patient’s “abnormal state of mind” could be traced back “to childhood” and, with the exception of menstruation, showed no “periodicity” (Jung, 1904, p. 28). The patient was skillful at school, was able to behave and had good grades, but was a lively child from an early age (Jung, 1904, p. 26). She was irritable at times and was repeatedly noted to be particularly “cheerful.” During her apprenticeship at the age of 16 years, the patient did little work, disobeyed her superiors and ultimately dropped out. According to Jung’s history, the patient generally showed “quick perception with little diligence” (Jung, 1904, p. 26) and was already conspicuous in her teenage years due to her erratic lifestyle. In addition to her frequent job changes, she was always busy and quickly bored. She seemed to be careless with money and quickly lost track of her finances, and often ran up debts.

*She had made no savings; she squandered what she earned and on top of that ran up debts everywhere (Jung, 1904, p. 27)*.

Jung describes the patient as “restless,” “*never calm, always busy and excited” (Jung, 1904, p. 27)* and often made rash decisions that she rejected the next moment. When the patient was admitted to the Burghölzli hospital in April 1903, she was diagnosed with “slight maniacal agitation” (Jung, 1904, p. 27). Jung summarized his observations as follows: “The findings: slight flight of ideas, talkativeness, predominantly elevated mood, instability, distractibility, urge to move, eroticism, confirm the diagnosis of manic excitement and explain the unsteady, morally defective way of life” (Jung, 1904, p. 28).

After presenting the four case descriptions, Jung talks about the similarities between the cases presented in the last section of his work and makes differential diagnostic considerations. It seems important to the young psychiatrist to emphasize that the patients described were all of good intelligence, which, however, stood in contrast to their “inexpedient” lifestyle (Jung, 1904, p. 36). According to Jung, a comparable contradiction between intelligence and general lifestyle would also be found in “*moral insanity.*”

The similarity to “moral insanity” seems essential for Jung here. He devotes an entire section to this and contemplates at length the relationship between intellect and will as well how actions are dependent on the mind. According to Jung, the decisive component lies in the patient’s “anomaly of mind” and is less related to their intellect in general.

In line with his claim to incorporate the psychology of the mentally ill into psychiatric considerations, Jung provides a detailed summary of the connection between intellect and feelings, as well as their influence on behavior. In his view, even the “purest intellectual process . . . [could] only reach the determination of the will by means of the emotional value. . .” (Jung, 1904, p. 38). Purely intellectual processes, that is rational thinking processes, are therefore ultimately determined by emotional values and feelings, so that when it comes to making a decision or carrying out an action the impact of feelings and hence also emotional excitation or lability may be the primary cause for inacceptable behavior. We could also conclude that emotions influence how we use our intelligence and act. Therefore, “given that the mind is largely intact, for every abnormal behavior, one should look for the primary cause in the affective area” (Jung, 1904, p. 38), as the mind or a person’s intelligence are not the determining factors for such behavior. According to Jung, it is patients with a manic (mood) disorder that often show such inadequate behavior. For Jung this affect defect or deficiency is typical for this group of patients and represents the relevant quality of this disease. He sees himself in agreement with Wernicke who also placed moral insanity “in a distant parallel to mania.” In conclusion, he emphasizes that even in the cases of “moral insanity” known to him, “emotional excitability and instability” are reported frequently (Jung, 1904, p. 38). Accordingly, when examining such patients Jung recommended putting more of a focus on to these mood abnormalities in order to possibly “put these cases in a different light” (Jung, 1904, p. 39). In other words, one should go beyond seeing their behavior as solely unacceptable or at least inadequate from an ethical point of view, and rather consider that it could be a “mood-related inferiority” by which Jung meant a “mild or severe manic mood” (Jung, 1904, p. 39).

Jung summarizes his observations on manic mood as follows: “1. manic mood is a clinical picture belonging to the field of psychopathic inferiority, which is characterized by a stable, submanic symptom complex that usually reaches back to adolescence. 2. exacerbations of uncertain periodicity occur. 3. alcoholism, criminality, moral insanity, social instability or incapacity are in this case symptoms dependent on the submanic state” (Jung, 1904, p. 39).

## Discussion

In light of the entrenched view of ADHD as a “fashionable diagnosis,” the question arises as to whether previous generations of psychiatrists also saw such patients in their day-to-day work, and if so, how they were categorized.

The work of Carl Gustav Jung offers an interesting insight in this context. In his case descriptions, he presents a detailed account of individual patient biographies which, from today’s perspective, seem typical of ADHD sufferers. He himself categorizes the cases of “manic maniacs” in the “area of psychopathic inferiorities” (Jung, 1904, p. 39). While German psychiatrist Julius Ludwig August Koch (1841–1908) coined this term toward the end of the 19th century as a generic term for abnormal personalities (Schmiedebach, 2016), the term was used synonymously with “mentally ill” for a long time. Koch’s definition was broad and included both eccentric behaviors and peculiarities as well as conditions that would be classified as neuroses today. He regarded these “psychopathic inferiorities” as congenital, persistent personality disorders that did not necessarily lead to severe mental illness but could promote deviant behavior and social problems (Koch, 1891). At the time, Psychiatry sought to explain undesirable behaviors in children and adults through the concept of “psychopathies.” It was often assumed that a genetic or intrauterine predisposition resulted in a “congenital inferiority” in affected individuals (Horley, 2014).

In the English-speaking world, the physician James Cowles Prichard (1786–1848), among others, had previously coined the term “moral insanity,” which also entered German psychiatric terminology. This term encompasses mental disorders that characterized by the absence of delusions, hallucinations, and intellectual disability (Augstein, 1996). Prichard developed this concept to describe individuals who, despite being intellectually unimpaired, exhibited impulsive, erratic, or antisocial behavior, without the presence of delusions, hallucinations, or other obvious mental illnesses. He thus introduced an early form of personality pathology, focusing on “moral” judgment and behavior. This construct was significant in relation to criminality and deviant behavior at the time (Augstein, 1996). Prichard’s concept of “moral insanity” was therefore an early attempt to categorize behavioral disorders that could not be explained by classical mental illnesses. Similarly, Koch expanded the understanding of mental disorders with his idea of “psychopathic inferiority,” emphasizing subtle yet profound personality deviations. Although the terms “moral insanity” and “psychopathic inferiorities” are no longer in use today, they were common, albeit inconsistent, diagnoses in the 19th and early 20th centuries (Lorettu et al., 2017; Steen, 1913). These concepts provided psychiatrists of that time with a way to categorize complex behavioral disorders and reflect the uncertainty in the psychiatric field regarding the precise classification of such phenomena.

The discourse in German psychiatry around and after 1900 regarding the construct of “psychopathy” was very diverse, vaguely defined, and by no means universally accepted in the psychiatric community. This is clearly reflected in the discussion about the “chronic-manic concepts” (Müller et al., 2024). In this context, Jung also addresses the “mixture” of “mild manic symptoms” with “other psychopathic traits” (Jung, 1904, p. 18). Jung is unsurprised by this, as it is his opinion that “the boundary between the clinical pictures in the area of psychopathic inferiority is extraordinarily indeterminate and fluctuating” (Jung, 1904, p. 18). The fundamental question of boundaries between the normal and the pathological, which goes hand in hand with the question of the boundary between individual images of pathology, also seems to have occupied Jung intensely during his reflections on how to classify the “manically disturbed.” As these conditions were placed within the construct of “psychopathies”—a framework that viewed illness and health as a continuum—it becomes evident that members of the medical profession in the early 20th century also faced uncertainties regarding the significance of individual pathological phenomena.

Even today, symptoms typical of ADHD are often attributed to other psychiatric syndromes. This observation can be explained by the fact that the core symptoms also occur in other disorders, such as affective disorders. Another reason could be that ADHD rarely occurs in isolation and the majority of patients has an additional comorbid psychiatric disorder (Hartman et al., 2023), which often leads to under- and misdiagnosis (Ginsberg et al., 2014) and a resulting lack of specific treatment. Jung also identifies this risk in patients with “manic mood.” He recommends looking at the patient cases previously categorized as “moral imbecility” from a new perspective (with regard to the “anomaly of the mind”) in order to “put one or the other case” in a different “light” (Jung, 1904, p. 39).

The patient biographies presented by Jung suggest that the “manic maniacs” at that time exhibited relevant functional limitations in all areas of life, similarly to ADHD sufferers today, which were noticed by those around them. Jung succeeds in tangibly conveying the various functional limitations in the respective areas of life of those affected. Subjective impairment is also an important criterion for the current diagnostic assessment of adult ADHD (American Psychiatric Association, 2022; Faraone et al., 2021). In his classification of causal chains, Jung’s assessment that “alcoholism, criminality, moral insanity, social instability or incapacity [. . .] are symptoms dependent on the submanic state” (Jung, 1904, p. 39) is remarkably consistent with current understandings of ADHD-associated comorbidities and adverse psychosocial outcomes. ADHD patients show an increased prevalence of addictive disorders (Groenman et al., 2017), violate laws more frequently (Mannuzza et al., 2008), and are more likely to have a low socioeconomic status and a low level of education as a result of unstable teaching and employment (Chen et al., 2024).

In his description of the “manic mood,” there are clear parallels to the ADHD psychopathology described today as typical (Table 2). In addition to the currently defined core symptoms of ADHD (attention deficit, hyperactivity, and impulsivity), Jung sees and describes a number of other symptoms in his patients that are also considered typical of ADHD today.

**Table 2.** Comparison Between Core ADHD Symptoms Defined Today and the Case Reports of C. G. Jung (1904).

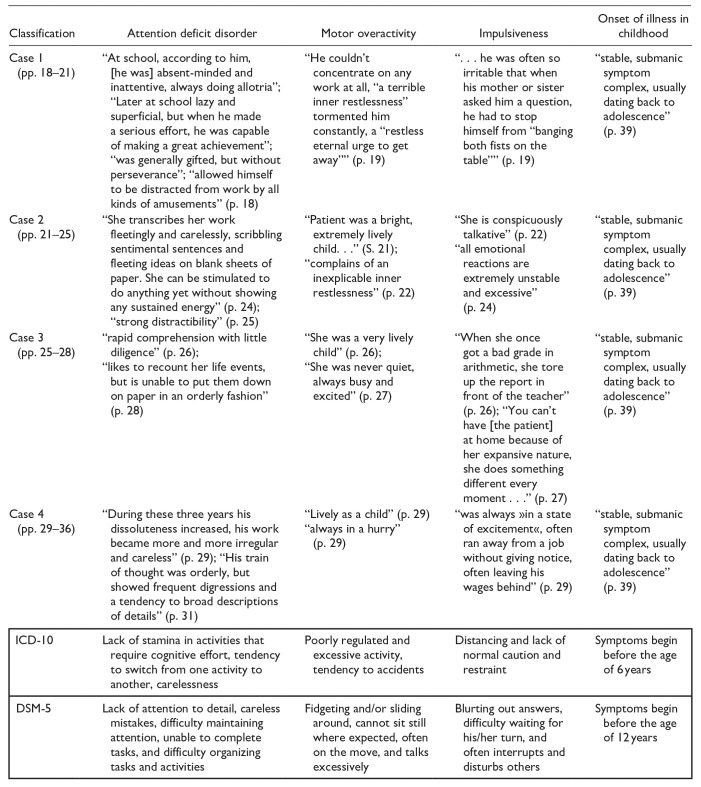

*Note.* *DSM-5* *=* Diagnostic and Statistical Manual of Mental Disorders 5; *ICD-10* *=* International Statistical Classification of Diseases and Related Health Problems 10. The highlighted table cells contain direct excerpts from Jung’s case studies, illustrating ADHS-related behavioral patterns observed in his patients. These passage demonstrate how Jung describe key ADHS symptom domains – such as inattention, impulsivity, and hyperactivity – using the clinical terminology of his time.

Jung recognizes that the clinical picture goes back “as far as adolescence” (Jung, 1904, p. 39) which suggests an onset of the disorder in adolescence. The patients mentioned in his case descriptions showed abnormalities at school at an early age, which Jung considered relevant. Even today, the onset of symptoms in the childhood is a key diagnostic criterion for ADHD (American Psychiatric Association, 2022). Jung’s explanation of the relationship between affect intolerance in the group of patients he described is noteworthy. Patients would exhibit “exacerbations of uncertain periodicity” and would often be emotionally unstable (Jung, 1904, p. 39). One of his patients “was easily moved to tears” (Jung, 1904, p. 18) and often “disgruntled for no reason, partly irritable, partly depressed” (Jung, 1904, p. 19). In another patient, Jung emphasizes the “great instability of pleasure and displeasure” (Jung, 1904, p. 25). Jung’s descriptions are strongly reminiscent of ADHD-associated emotional dysregulation (Beheshti et al., 2020), which has been described as an additional core symptom of the disorder (Hirsch et al., 2018).

In the published case reports, it is also noticeable that, on the one hand, those affected stood out with attention and concentration deficits within their school and professional lives, but were also able to devote themselves intensely and persistently to some tasks. For example, Jung reports on a patient who was “passionately fond of reading novels (often for half the night)” (Jung, 1904, p. 26) or about another patient who, despite his absent-mindedness and inattentiveness, was capable of “making a great achievement . . . provided that he made a serious effort” (Jung, 1904, p. 18). This phenomenon of hyperfocus is also well documented in people with ADHD (Hupfeld et al., 2019).

ADHD sufferers also often have a poor relationship with money and finances. Due to a deficient ability to self-regulate and increased impulsivity, many ADHD patients easily lose track of their finances and tend to make frequent impulse purchases (Bangma et al., 2019). Jung also reports similar observations in one of his cases: “She had made no savings; she squandered what she earned and, on top of that, ran up debts everywhere” (Jung, 1904, p. 27).

Finally, reference should be made to Jung’s differentiation of the clinical picture from bipolar disorder. Jung states that the “manic mood” was by no means “an actual mania, but merely a submanic state.” The “relatively mild manic symptoms [were] not partial manifestations of a periodic mania” (Jung, 1904, p. 18). Elsewhere, he adds that there is no “periodicity” in the symptoms described above (Jung, 1904, p. 28). From today’s perspective, these observations are highly relevant for the differential diagnosis of the current concept of bipolar disorder and can be used as a further argument to support that ADHD patients were among Jung’s listed patient cases.

With his work, Jung makes an important contribution to the early 20th-century debate on the existence of chronic mania. The impact of this discourse on subsequent generations of psychiatrists is difficult to assess but, in retrospect, seems to have had little direct influence on the history of ADHD. This may be due to the fact that the psychopathology typical of ADHD remained outside the scope of adult psychiatry for a long time, and Jung’s “manically disordered “ patients may not have received sufficient attention. Another reason could be that these patients—unlike those with severe psychoses or major affective disorders—were able to organize their daily lives relatively well, were not entirely incapacitated, and therefore were far less frequently treated as inpatients in clinics. Later “ADHD research” in the following years heavily focused on behavioral abnormalities in children. With the first descriptions of post-encephalitic behavioral disorders, the prevailing view temporarily emerged that subtle brain damage could be the cause of ADHD-like symptoms. Subsequently, the two German psychiatrists Franz Kramer (1878–1967) and Hans Pollnow (1902–1943) reported on a “hyperkinetic disease of childhood,” which from today’s perspective is considered a key reference point for the early conceptualization of ADHD under the name of the “Kramer-Pollnow Syndrome” (Neumärker, 2005). Further works by Eugen Kahn (1895–1961) and Louis H. Cohen (1897–1983; Kahn & Cohen, 1934) citing some adult examples reinforced the prevailing assumptions of a causal “minimal brain damage” (Kessler, 1980). However, in the second half of the 20th century, research gradually moved toward a more nuanced understanding of ADHD, increasingly including adults as well. Jung, on the other hand, did not return to the topic of the “manically disordered” in the following years and instead turned toward analytical psychology, the interest for which he is best known. It is possible that many of his chronically manic patients were later subsumed into the field of psychopathy, making a closer analysis of early 20th-century psychopathy theories potentially suitable for further illuminating the origins of ADHD (Table 3).

**Table 3.** Explanatory Table of Key Historical Terms in This Paper.

Term	Definition and context
Inferiority	The term “Inferiority” (from the German *Minderwertigkeit*) was historically used in 19th- and 20th-century psychiatry to describe perceived psychological, emotional, and moral deficits. It was often associated with psychopathic personalities or psychopathological phenomena, focusing on perceived impairments in personality or behavior. *cf. psychopathic inferiority*
Instability	A term historically used to describe emotional or psychological variability, often linked to behavioral unpredictability.
Moral insanity	Introduced by James Cowles Prichard (1786–1848) in 1835, this term described a pathological condition characterized by a lack of honor, remorse, and conscience. Prichard categorized it under combined disorders of emotions (“disorders of affection or feeling”) and volition (“those of the active powers or propensities”). Defined as “a disease consisting of a morbid perversion of the natural feelings, affections, inclinations, temper, habits, and natural impulses, without any noticeable disorder of intellect, memory, or judgment, and particularly without hallucinations or delusions” (Prichard, 1835, p. 10). Moral insanity remained a recognized diagnostic entity until the 20th century (Peters, 2004).
Psychopathy	A historical term used to describe personality disorders characterized by antisocial behavior and emotional deficits. Julius Ludwig August Koch (1841–1908), a German psychiatrist, coined this term in the late 19th century as a generic term for abnormal personalities (Schmiedebach, 2016). For decades, it was synonymous with “mentally ill.” Koch’s broad definition included eccentric behaviors and peculiarities as well as conditions resembling today’s neuroses. He described these “psychopathic inferiorities” as congenital and persistent personality disorders that did not necessarily impair intellect but could promote deviant behavior and social difficulties (Horley, 2014; Koch, 1891).
Psychopathic Inferiority	A term introduced by Julius Ludwig August Koch (1841–1908) in his 1888 *Short Textbook of Psychiatry (Kurzgefasster Leitfaden der Psychiatrie (Koch, 1891))*. “*Psychopathic inferiority*” *(psychopathische Minderwertigkeiten)* referred to inherent psychological or moral deficiencies that did not impair intellect but significantly affected emotional stability and social behavior. cf. *Psychopathy and Inferiority*
Sanguine temperament	One of the four classical temperaments derived from Hippocrates’ theory of types, describing individuals who are lively, cheerful, carefree, and spirited.
Unstable people in Magnan’s sense	Referring to the French psychiatrist Jacques Joseph-Valentin Magnan (1835–1916), a key figure in degeneration theory during the *Fin de Siècle* (Shorter, 2010).

## Conclusion

In summary, Carl Gustav Jung’s concept of “manic mood,” described in 1904, has clear parallels to the diagnostic criteria commonly used for adult ADHD. It is conceivable that the patients presented by Jung would be diagnosed with adult ADHD today. Although Jung provides a detailed description of the characteristics of the “manically disordered” and differentiates them from other disease entities while emphasizing a multidimensional approach to psychiatry, a critical assessment must note that Jung did not comment on the actual cause or ultimate treatment of such “manically disordered” patients in his work. Considering the potential danger of viewing the analyzed material too much in isolation, Jung’s work fits into the discussion of “chronic-manic concepts” of the early 20th century and thus makes a relevant original contribution to the nosological-diagnostic classification of forms in the spectrum of ADHD-affective disorders-personality disorders. A definitive classification within these frameworks cannot be established based on the final analysis. While the similarities between the cases described by Jung and the modern understanding of ADHD are evident, affective components also appear to play a significant role. This indicates that Jung’s descriptions cannot be exclusively attributed to ADHD. Although the two concepts are not entirely congruent, Jung’s case studies nevertheless provide detailed, partially “ADHD-typical” descriptions of patients and incorporate aspects into his clinical discussions that align with the core symptoms of adult ADHD as understood today and with current research questions. The identified parallel could thus represent another important cornerstone in reconstructing the conceptual history of adult ADHD around 1900. The fact that concepts like “chronic mania,” to which Jung contributed, were considered pathological by the German-speaking medical community around 1900 suggests that the perception of ADHD-typical symptoms as a disorder is not new but has been established as a pathological phenomenon over a longer historical timeline. This demonstrated conceptual flexibility is reflected in evolving terms and classifications. At the same time, it shows remarkable stability in its recognition as a pathological phenomenon, evident in both its temporal continuity and the repeated conceptual engagement with similar syndromes. These varying conceptualizations may underscore the complexity and ambiguities that persist in the international context even today. Against this backdrop, dismissing ADHD as a mere “fad diagnosis” appears overly reductive and inappropriate.

While Jung did not revisit the topic of “manic mood” as pointedly as in the work presented here, further research into the history of psychiatry which examines the aforementioned intersection of the “psychopathy doctrine” at the beginning of the 20th century in more detail, could be suitable to further substantiate our hypothesis.
